# Supplementary material for: Mathematical modeling and simulation of tumor-induced angiogenesis in retinal hemangioblastoma
Source: PLoS Comput Biol. 2025 Sep 11;21(9):e1012799. doi: 10.1371/journal.pcbi.1012799 (PMC12503348; doi:10.1371/journal.pcbi.1012799)
Supplement: S1 Text — Fig A in S1 Text. Comparison between Fundus Angiography and OCTA. Large RH reported by Sagar P. and collaborator [67]. a) Fundus Angiography shows leakage and exudation around the tumor but does not allow a clear observation of the tumor borders and of the capillaries. b) OCTA image displays tumor borders, high vascularity, and major blood vessels enlargement and tortuosity. Fig B in S1 Text. AF distribution in space and time. Upper panels: AFs distribution at time 0, considering the simulations in Fig 2 of the manuscript. The contour of the tumor shape is shown in yellow. Lower panels: mean AFs concentration (bold line) and interquartile range (light blue area) in time during the simulation. For each patient, we observe that the AFs are more concentrated inside the tumor. In time, there is a slight decrease in the mean AFs concentration. Fig C in S1 Text. First month of tumor-induced angiogenesis for P0. The pictures on the left (A, C, E) display the vascular development with Vuc=2.3·10−4 s-1, while the pictures on the right refer to the simulation for Vuc=17.8·10−4 s-1. In both the simulations we observe the formation of a stable vascular structure, which is bolder for the first case and thinner for the latter. G) and H) show the number of active tip cells throughout the simulations. Fig D in S1 Text. First month of tumor-induced angiogenesis for P1. The pictures on the left (A, C, E) display the vascular development with Vuc=2.3·10−4 s-1, while the pictures on the right refer to the simulation for Vuc=17.8·10−4 s-1. In the first simulation the initial vascular is not stable and slowly disappear in time, while in the second the novel capillary maintains its stability. G) and H) show the number of active tip cells throughout the simulations. Fig E in S1 Text. First month of tumor-induced angiogenesis for P2. The pictures on the left (A, C, E) display the vascular development with Vuc=2.3·10−4 s-1, while the pictures on the right (B, D, F) refer to the simulation fo [file pcbi.1012799.s001.docx]

Supporting Information for

Mathematical Modeling and Simulation of Tumor-Induced Angiogenesis in Retinal Hemangioblastoma

Franco Pradelli, Giovanni Minervini, Pradeep Venkatesh, Shorya Azad, Hector Gomez, Silvio C.E. Tosatto

Corresponding author: Silvio C.E. Tosatto

Email: [silvio.tosatto@unipd.it](mailto:silvio.tosatto@unipt.it)

**This file includes:**

Supporting text

Supporting Figures A to L

Supporting Tables A and B

SI References

Supporting Information Text

Section 1: Mathematical model

**Detailed mathematical model.** Our mathematical model is composed of the following equations:

| Retinal Hemangioblastoma (RH) Growth | *Equation s1*  $\varphi\left( \vec{\boldsymbol{x}},t \right)=\left\{ \begin{matrix} 1 & \mathrm{if} \left( \frac{x}{s_{x}\left( t \right)} \right)^{2}+\left( \frac{y}{s_{y}\left( t \right)} \right)^{2}+\left( \frac{z}{s_{z}\left( t \right)} \right)^{2}\leq1 \\ 0 & \mathrm{Otherwise} \end{matrix} \right.$  Where:  *Equation s2*  $s_{x}=s_{y}=\frac{d_{p}}{2}\mathrm{tg}r^{\frac{t}{3}}\quad\quad s_{z}=\frac{d_{a}}{2}\mathrm{tg}r^{\frac{t}{3}}$ |
| --- | --- |
| Capillaries PDE | *Equation s3*  $\frac{\partial c}{\partial t}=M\nabla^{2}\left[ -c+c^{3}-\epsilon\nabla^{2}c \right]+B_{p}\left( af \right)\mathrm{cH}\left( c \right)$  Where:  *Equation s4*  $B_{p}\left( \mathrm{af} \right)=\left\{ \begin{matrix} 0 & if af\leq0 \\ \alpha_{p}af & \mathrm{if} 0\leq af\leq af_{p} \\ \alpha_{p}af_{p} & if af>af_{p} \end{matrix} \right.$ |
| Tip Cells (TC) velocity | *Equation s5*  $v=\left\{ \begin{matrix} \chi\frac{\nabla af}{G} & \mathrm{if}G_{m}\leq G<G_{M} \\ \chi\frac{\nabla af}{G}G_{M} & if G\geq G_{M} \end{matrix} \right.$ |
| Tip Cells (TCs) and Stalk Cells (SCs) value | *Equation s6*  $c_{c}=\frac{S_{p}\left( af \right)\pi R_{c}}{2\left\vert v \right\vert}$  Where:  *Equation s7*  $S_{p}\left( \mathrm{af} \right)=\left\{ \begin{matrix} 0 & if af\leq0 \\ \alpha_{pSC}af & \mathrm{if} 0\leq af\leq af_{p} \\ \alpha_{pSC}af_{p} & if af>af_{p} \end{matrix} \right.$ |
| Angiogenic Factors (AFs) | *Equation s8*  $0=D_{\mathrm{af}}\nabla^{2}\mathrm{af}+V_{\mathrm{pT}}\cdot\varphi\cdot\left( 1-H\left( c \right) \right)-V_{\mathrm{uc}}\cdot af\cdot H\left( c \right)-V_{d}\cdot af$ |

Plus, there are algorithms taking care of tip cells’ (TCs) activation and deactivation, which we represented in Figure H.

Section 2: Parameters

Each of the equations and algorithms presented above depend on several parameters, which, when possible, were based on experimental evidence or independent estimations. In the following, we describe how we derived each parameter. Note that we often estimated the parameters regarding the angiogenic factors (AFs) using the vascular endothelial growth factor (VEGF) as the primary reference. Indeed, the association of this cytokine with tumor-induced angiogenesis is widely recognized [1]; moreover, there is experimental evidence on the fact that VEGF is overexpressed in RH [2].

On the GitHub repository related to this manuscript, you can also find a Jupyter Notebook to make the evaluation of each parameter fully reproducible.

**Arbitrary Units.** We choose 800 μm as space arbitrary unit (sau), which is of the same order of magnitude of the bigger RH diameters as observed in patients (see section **Initial RH** ).

As the time arbitrary unit (tau) we assumed 26 min, which is the time step value used to solve the PDE system. The same value is used by Travasso and collaborators in the original model [3] and allows the integration between the PFM model and the algorithm for TC. Indeed, given that the maximum TCs’ velocity (see sections $G_{m}$, $G_{M}$, and $\chi$ (Tip cells velocity)) is:

$v_{\max}=0.35$ μm·min^-1^

The maximum displacement of a TC in one time step is:

$s_{\max}=v_{\max}\cdot dt=9.1$ μm

Which is less than one TC radius (see section $R_{c}$ (Tip Cells’ radius)). In this implementation, using a higher value of dt could lead to a discontinuous $c$ field.

For AFs concentration, we assumed an arbitrary unit equal to 6 ng·mL^-1^. This value represents a relatively high concentration value. It was selected based on the maximum VEGF concentration reported by Na X. and collaborators [4], which measured VEGF concentration in some VHL-deficient kidney tumors.

**Initial RH dimension.** For each patient, we could estimate the RH diameters using the OCTA at different depths and the B scans. For patient 0 (P0) and patient 2 (P2) the tumor shape was clearly visible from the B-scan. For patient 1 (P1) we estimated the depth of the lesion being equal as the depth of the inner retina, as there was no sign of abnormal vascularization in the outer retina (see Deep OCTA for Patient 1 in Fig I). See Table B for a summary of the spatial features of the different RHs.

$\boldsymbol{tgr}$ **(Tumour growth rate).** The average growth rate for VHL-related hemangioblastoma has been reported to be 0.35% per year (considering the tumor volume's increase) [5]. Therefore, we employed this value for our model.

**M (Motility for c field).** This value represents the motility of the capillaries phase field $c$, which we assumed to be in line with the value used by Travasso et al. [3] in the original paper ($M={10}^{-15}$ m^2^·s^-1^). For the simulation in Figure 5 of the manuscript, we used a value that is 10 times lower.

$\boldsymbol{\epsilon}$ **(Interface width for the c field).** This value represents a measure of the stable interface’s width for the capillaries phase field c, which we assumed to be equal to the value used by Travasso et al. [3] in the original paper ($\epsilon^{2}={1.25}^{2}$ μm^2^).

$\boldsymbol{B}_{\boldsymbol{p}}$ **(Proliferation for mature endothelial cells) and** $\boldsymbol{S}_{\boldsymbol{p}}$ **(proliferation for stalk cells).** In the original model [3], there was no distinction between mature endothelial cells’ and stalk cells’ proliferation. They considered only proliferation due to stalk cells (SCs), for which they assumed:

$$\alpha_{p}\cdot af_{p}=0.97 \mathrm{hr}^{-1}\quad\quad\mathrm{af}_{p}=0.3 \mathrm{afau}\alpha_{p}=\frac{0.97}{0.3} \mathrm{hr}^{-1}\mathrm{afau}^{-1}$$

Which represents the maximum proliferation for the SCs. Notice that this value represents an increase in volume per unit time and not an exact proliferation rate for SCs; however, its choice is based on *in vitro* evidence, as justified by Travasso and collaborators [3]. Thus, we kept the same parameters values for SCs’ proliferation:

$$\alpha_{pSC}\cdot af_{p}=0.97 \mathrm{hr}^{-1}\quad\quad\mathrm{af}_{p}=0.3 \mathrm{afau}\alpha_{pSC}=\frac{0.97}{0.3} \mathrm{hr}^{-1}\mathrm{afau}^{-1}$$

However, for mature endothelial cells, we neglected the proliferation induced by AFs, assuming:

$$\alpha_{p}=0$$

This choice has two reasons. First, performing simulations with several $\alpha_{p}$ values (e.g., $=\alpha_{pSC}$, $=\frac{\alpha_{pSC}}{10}$), we observed capillaries enlargement instead of sprouting angiogenesis, demonstrating that our model was not correctly reproducing the clinical images for these parameter choices (see an example in Figure J). Second, mature blood vessels are more stable than novel capillaries, and the proliferation of mature endothelial cells follows different rules, which we choose not to cover in our model [6]. Thus, it is reasonable to assume that mature endothelial cells’ proliferation is negligible compared with SCs’ proliferation.

$\boldsymbol{G}_{\boldsymbol{m}}$**,** $\boldsymbol{G}_{\boldsymbol{M}}$**, and** $\boldsymbol{\chi}$ **(Tip cells velocity).** Equation s5 defines the TCs’ velocity range, which has been measured both *in vitro* and *in vivo*. Travasso and collaborators [3] assumed the maximum value for TCs’ velocity to be:

$$v_{max}=0.35 \mu m\cdot{min}^{-1}$$

This value was derived from experimental evidence *in vitro* [7]. Moreover, it agrees with the TCs’ velocity measured during sprouting angiogenesis [8], and *in vivo* experiments [9,10].

$G_{m}$ represents the minimum value of G necessary to activate the tip cells. The lowest VEGF gradient triggering endothelial cells’ migration we could find in the literature is 14 ng·nL^-1^·mm^-1^ [11]. Thus, we assumed:

$$G_{m}=14 ng\cdot\mathrm{mL}^{-1}\cdot\mathrm{mm}^{-1}$$

Then, coherently with the original model, we assumed $G_{M}={3G}_{m}$:

$$G_{M}=42 ng\cdot\mathrm{mL}^{-1}\cdot\mathrm{mm}^{-1}$$

This leads to the assumption that TCs’ velocity linearly increases with G in the range [14, 42] ng·mL^-1^·mm^-1^. The fact that TCs’ velocity depends on G over a limited range has been proved experimentally by Barkefors I. and collaborators [12]. They also provide approximate G values necessary for endothelial cells’ migration, which are slightly higher but in agreement with ours (about [50, 100] ng·mL^-1^·mm^-1^).

Finally, we assume that $\chi$ is such to limit the maximum TCs’ velocity to $v_{max}$, so:

$$\chi=\frac{v_{max}}{G_{M}}$$

$\boldsymbol{R}_{\boldsymbol{c}}$ **(Tip Cells’ radius).** Assuming the value provided on the BioNumbers database (Entry ID: 100432; [13]), we have:

$$R_{c}=10 \mu m$$

$\boldsymbol{T}_{\boldsymbol{c}}$ **(Minimum AFs concentration for tip cells activation).** An independent estimation conducted by Song et al. [14], states that endothelial sprouting starts for VEGF values around 3 ng / mL. Thus, we set:

$$T_{c}=3 ng\cdot\mathrm{mL}^{-1}$$

$\boldsymbol{\delta}_{\boldsymbol{4}}$ **(Minimum TCs distance due to the Notch pathway).** Coherently with the original model [3], we assume that the Notch pathway prevents the activation of two neighbor cells. Thus, we assume the minimum TC distance to be equal to $4R_{c}$:

$$\delta_{4}=4R_{c}=40 \mu m$$

$\boldsymbol{D}_{\boldsymbol{af}}$ **(AFs diffusivity).** Even considering VEGF alone, we could not find an agreed diffusivity value among different publications. VEGF diffusivity has been estimated in several publications (see Table S1) with values ranging from ${10}^{-7}$ to ${10}^{-4}$ mm^2^·s^-1^.

Thus, we derived our own estimation based on the experiments reported by Kihara et al. [15].

In [15], the authors measured the diffusivity of different biomolecules with different molecular weights. Among the different molecules, they tested Alexa488-dextran and FITC-dextran, which have molecular weights of 10 kDa and 40 kDa respectively. Given that the different isoforms of VEGF have a molecular weight included in the range [16, 45] kDa (derived from UniProt [16]; identifiers P15692-10 and P15692-14), we assumed VEGF diffusivity to be in the same range.

More precisely, considering the average molecular weight of 30.5 kDa, we can find an estimation of VEGF diffusivity by interpolating the two values for Alexa488-dextran and FITC-dextran:

$$D_{\mathrm{VEGF}}\cong\frac{D_{\text{FITC-dextran}}-D_{\text{Alexa488-dextran}}}{40-10}\cdot\left( 30.5-10 \right)+D_{\text{Alexa488-dextran}}$$

Resulting in:

$$D_{\mathrm{VEGF}}=D_{\mathrm{af}}=4.24\cdot{10}^{-5} {mm}^{2}\cdot s^{-1}$$

$\boldsymbol{V}_{\boldsymbol{pT}}$ **(AFs production rate inside the tumor).** Tumor VEGF secretion has been measured *in vitro* and has been estimated *in vivo* by Finley S. and collaborators [17]. They estimated VEGF secretion *in vivo* to be in the range $\left[ 0.007,0.023 \right]$ molecules / (cell·s), while *in vitro* measures have evaluated it to be in the range $\left[ 0.03, 2.65 \right]$ molecules / (cell·s).

Since an impaired VEGF regulation often characterizes von Hippel-Lindau-related tumors, we assumed the VEGF production rate to include both the in vitro and the in vivo range, so to have $V_{pT}\in\left[ 0.007, 2.65 \right]$ molecules / (cell·s).

To convert this range to pg·mL^-1^·s^-1^, we considered the VEGF molecular weight as registered on UniProt [16], which reports 16 kDa for the smallest VEGF isoform (identifier: P15692-10) and $45 kDa$ for the biggest (identifier: P15692-14). Considering this as the weight of one mole of VEGF, we have:

$$V_{pT_{min}}=\frac{16\cdot{10}^{3}}{c_{Avogadro}}\cdot0.007=0.02\cdot{10}^{-20} \frac{g}{cell\cdot s}$$

$$V_{pT_{max}}=\frac{45\cdot{10}^{3}}{c_{Avogadro}}\cdot2.65=19.8\cdot{10}^{-20} \frac{g}{cell\cdot s}$$

Then, we estimated the cell density per unit volume (CD). Assuming a tumor cell to have the same volume as a sphere of radius $R_{c}$, we have that one volume unit full of tumor cells contains:

$$CD_{max}=\frac{1}{\frac{4}{3}\pi R_{c}^{3}}\cong2.4\cdot{10}^{-4} \frac{\mathrm{cells}}{\mu m^{3}}\cong2.4\cdot{10}^{8} \frac{\mathrm{cells}}{\mathrm{mL}}$$

If we want to be more conservative, we can assume that only 75% of space in the capillary wall is composed of cells, while the rest is the extracellular matrix. This leads to a slightly lower estimation:

$$CD_{min}=\frac{0.75}{\frac{4}{3}\pi R_{c}^{3}}\cong1.8\cdot{10}^{-4} \frac{\mathrm{cells}}{\mu m^{3}}\cong1.8\cdot{10}^{8} \frac{\mathrm{cells}}{\mathrm{mL}}$$

Thus, we have:

$$V_{pT_{min}}=1.8\cdot{10}^{8}\cdot0.02\cdot{10}^{-20}=0.036 \frac{\mathrm{pg}}{mL\cdot s}$$

$$V_{pT_{max}}=2.4\cdot{10}^{8}\cdot19.8\cdot{10}^{-20}=47.5 \frac{\mathrm{pg}}{mL\cdot s}$$

Finally, the conversion to afau·tau leads to the range:

$$V_{pT}\in\left[ 0.0087,12.3 \right] \frac{\mathrm{afau}}{\mathrm{tau}}$$

$\boldsymbol{V}_{\boldsymbol{uc}}$ **(AFs uptake by the capillaries).** The AFs uptake factor represents the sum of the biophysical phenomena which result in AFs draining from the lesion. The blood flow plays a critical role in AFs transport, but other factors, such as AFs’ receptors uptake and platelets uptake, cannot be neglected at such a small scale. Thus, it is hard to find an experimental reference for the value (or range) of $V_{uc}$.

Thus, we used different reasoning to estimate a range for this parameter. First, we assumed that $V_{uc}$ must be equal or higher than the minimal natural degradation rate for VEGF, $V_{d}$ (see section $V_{d}$ (AFs degradation factor)):

$$V_{uc_{min}}=0.83 \mathrm{hr}^{-1}=2.3\cdot{10}^{-4} s^{-1}$$

To estimate the maximum value, we selected the value leading to no TCs activation for any patient and any value of V_pT_ (see Results in the main text). Indeed, the clinical images we selected display only highly vascular tumors, making, any $V_{uc}$ value not triggering sprouting angiogenesis not interesting for our study.

The selected value was obtained increasing of 10000 times $V_{{uc}_{min}}$:

$$V_{uc_{max}}=2.3 s^{-1}$$

$\boldsymbol{V}_{\boldsymbol{d}}$ **(AFs degradation factor).** According to Vempati et al. [18] the VEGF degradation rate is in the range $\left[ 0.83,1.008 \right]$ hr^-1^. Thus, we assumed the average $0.918$ hr^-1^ as reference value and the minimum as $V_{uc_{min}}$ (see previous section).

Section 2: Methods

**3D Reconstruction Algorithm (RA).** We depicted a schematic representation of our RA in Figure L. The output of RA is a Phase-Field, i.e., a 3D scalar field which equals 1 inside the capillaries and -1 outside. Thus, each mesh point must be mapped to 1 or -1 accordingly. For brevity, we will refer to this scalar field as 3D-PICN.

The algorithm works with the following inputs:

- The 2D segmentation of the putative initial capillaries network (2D-PICN)
- The distance transform of the segmentation. This is a matrix containing a skeletonized version of the 2D-PICN, where each pixel of the skeleton has the value of the local width of the vessel in that specific point (see Fig K).
- The mesh where the vessels should be reconstructed.
- The height of the vessels in the mesh (z0).

The procedure consists of 3 main steps:

1. *Rescaling 2D-PICN*

In this step, the 2D-PICN is rescaled. The output is a 2D scalar field equal to 1 inside the capillaries and to -1 outside (see Figure LA).

1. *Setting 2D-PICN to z0*

Then, we assumed that the 2D-PICN represents a section of 3D-PICN at a given height, z0. Thus, the second step sets the mesh points at height z0 equal to the points of the rescaled 2D-PICN. In our case, we assumed z0 to be 80 μm under the retinal surface for each patient, because only the superficial layer of the retina is vascular.

1. *Mapping all other points to 1 or -1.*

All other mesh points are mapped to 1 or -1 according to the algorithm in Figure LB. For every given point P, the algorithm works as follows:

- 1. It computes the projection of the point P on the plane z0 (P_z0_) and determines its value based on the rescaled 2D-PICN. If the value at P_z0_ is -1, the point P cannot be inside a capillary in 3D. Thus, the algorithm maps P to -1 and stops immediately. Notice that a projection of a point P = (x_P_, y_P_, z_P_) is computed simply by changing z_P_ to z0, so to have: P_z0_ = (x_P_, y_P_, z0).
  2. If P_z0_ is 1, the algorithm computes the Closest Center Point (CCP), which is the closest mesh point belonging to the skeleton of 2D-PICN (i.e., the closest point where the distance transform is higher than 0).
  3. Once the CCP is known, the rule is simple: if the distance between P and CCP is bigger than the value encoded for that point in the distance transform (DT VAL), then P is mapped to -1. Else, P is mapped to 1. This rule implies that the DT VAL encodes the information on the local radius of the vessels, and reconstruct cylindrical vessels, with a section close to the local section on 2D-PICN.

The result of the reconstruction for each patient is depicted in Fig. 2 and Fig 4 of the manuscript.


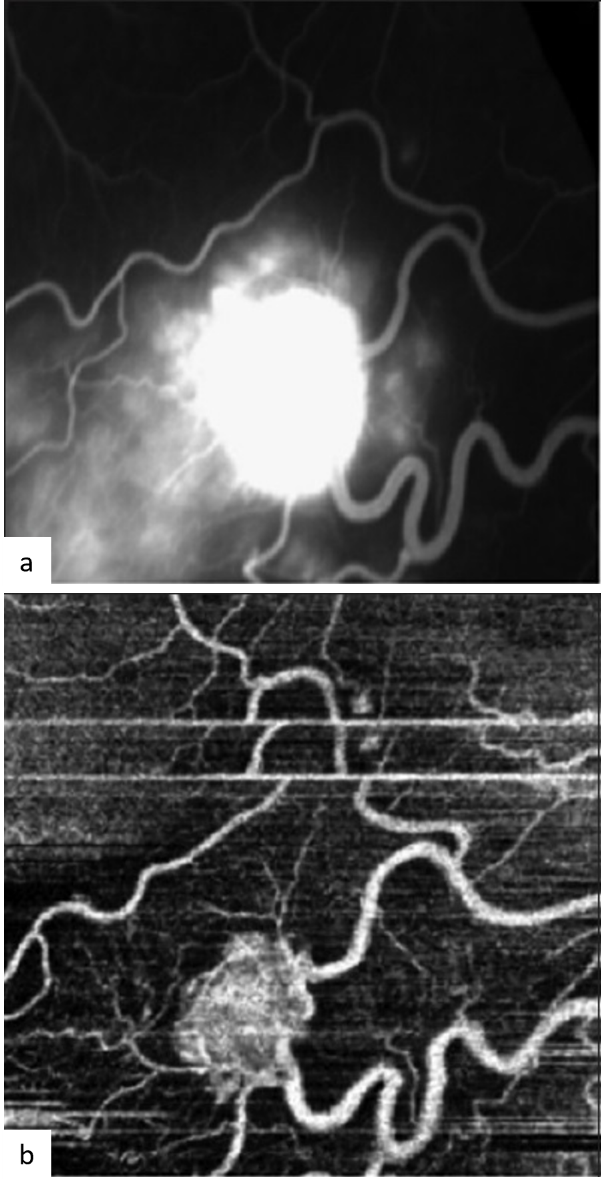


**Fig A. Comparison between Fundus Angiography and OCTA.** Large RH reported by Sagar P. and collaborators [19]. a) Fundus Angiography shows leakage and exudation around the tumor but does not allow a clear observation of the tumor borders and of the capillaries. b) OCTA image displays tumor borders, high vascularity, and major blood vessels enlargement and tortuosity.


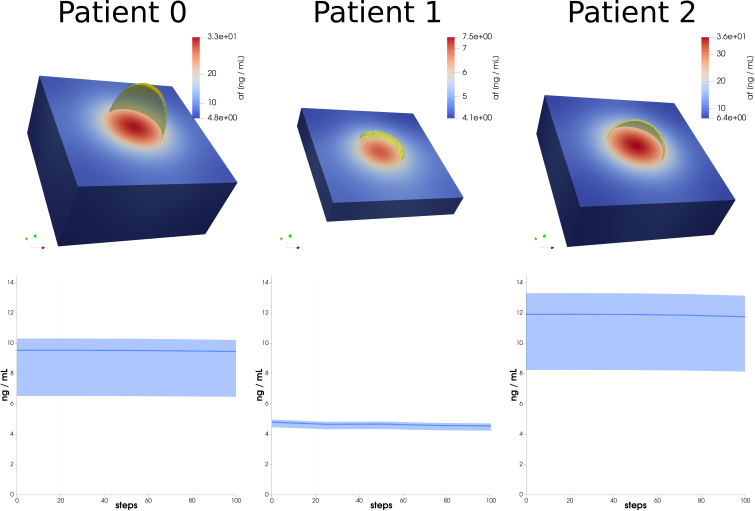


**Fig B.** **AF distribution in space and time.** Upper panels: AFs distribution at time 0, considering the simulations in Fig. 2 of the manuscript. The contour of the tumor shape is shown in yellow. Lower panels: mean AFs concentration (bold line) and interquartile range (light blue area) in time during the simulation. For each patient, we observe that the AFs are more concentrated inside the tumor. In time, there is a slight decrease in the mean AFs concentration.


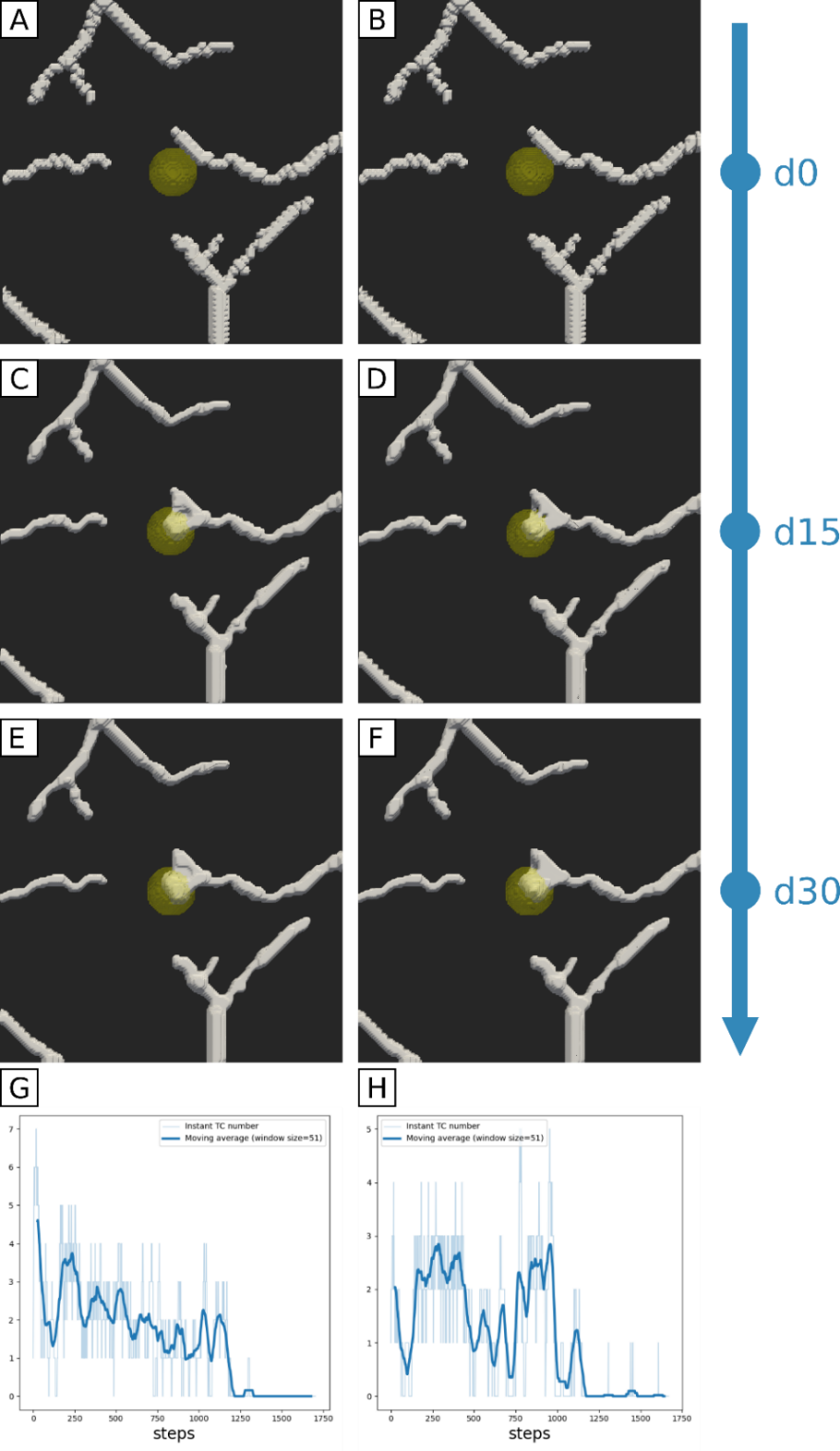


**Fig C. First month of tumor-induced angiogenesis for P0.** The pictures on the left (A, C, E) display the vascular development with $V_{uc}=2.3\cdot{10}^{-4}$ s^-1^, while the pictures on the right refer to the simulation for $V_{uc}=17.8\cdot{10}^{-4}$ s^-1^. In both the simulations we observe the formation of a stable vascular structure, which is bolder for the first case and thinner for the latter. G) and H) show the number of active tip cells throughout the simulations.

**
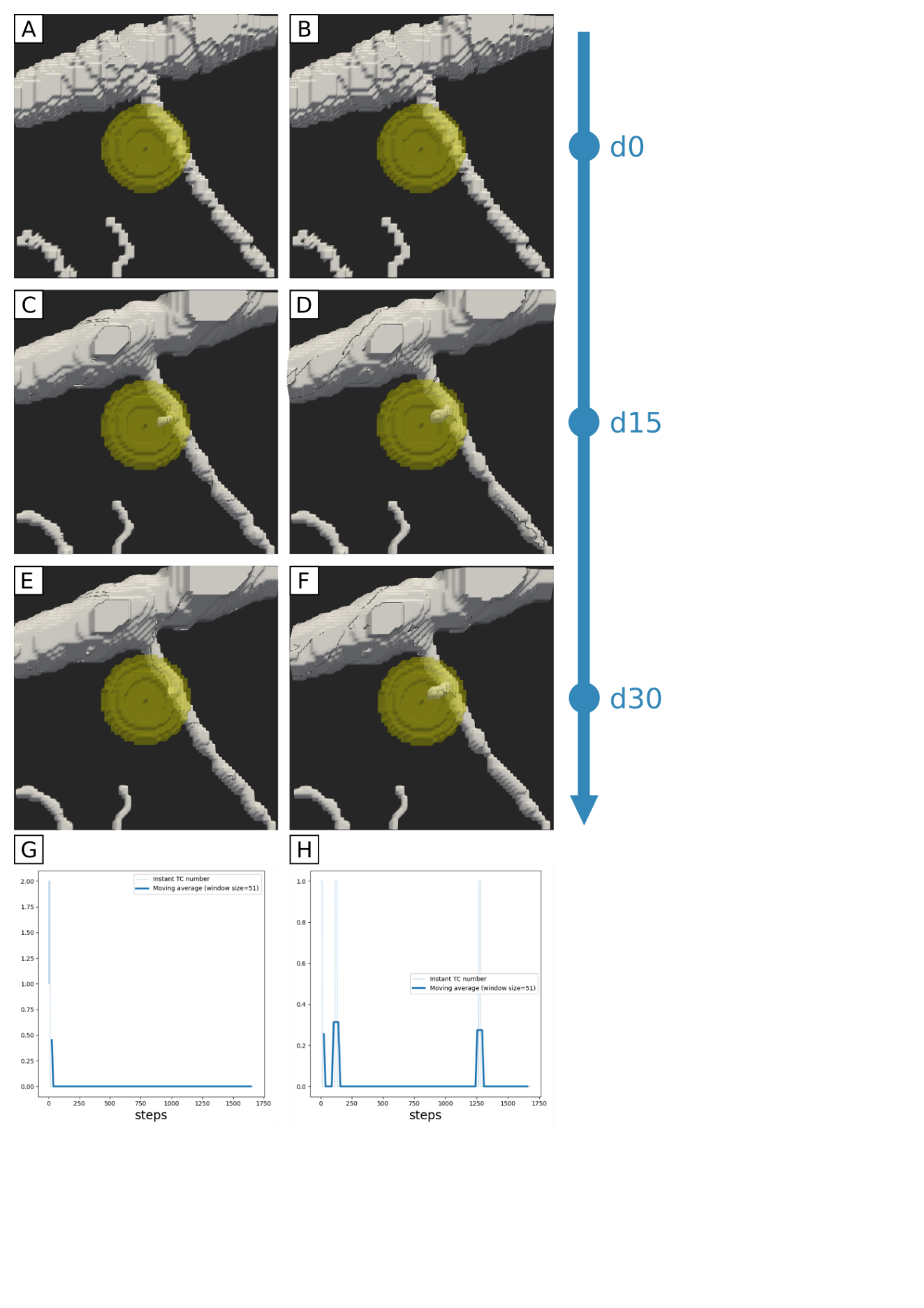
**

**Fig D. First month of tumor-induced angiogenesis for P1.** The pictures on the left (A, C, E) display the vascular development with $V_{uc}=2.3\cdot{10}^{-4}$ s^-1^, while the pictures on the right refer to the simulation for $V_{uc}=17.8\cdot{10}^{-4}$ s^-1^. In the first simulation the initial vascular is not stable and slowly disappear in time, while in the second the novel capillary maintains its stability. G) and H) show the number of active tip cells throughout the simulations.


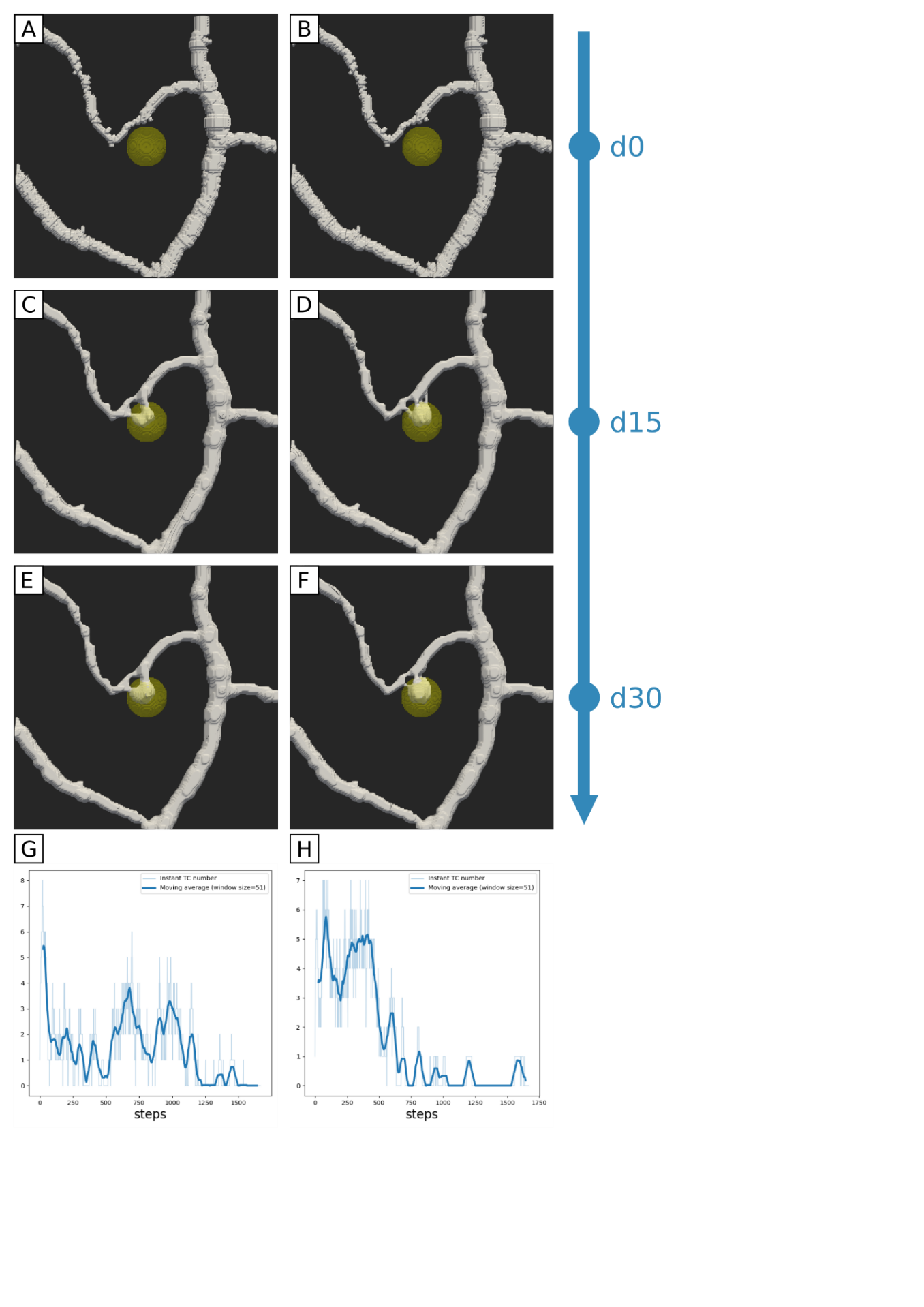


**Fig E. First month of tumor-induced angiogenesis for P2.** The pictures on the left (A, C, E) display the vascular development with $V_{uc}=2.3\cdot{10}^{-4}$ s^-1^, while the pictures on the right (B, D, F) refer to the simulation for $V_{uc}=17.8\cdot{10}^{-4}$ s^-1^. In both the simulations we observe the formation of a stable vascular structure. G) and H) show the number of active tip cells throughout the simulations.


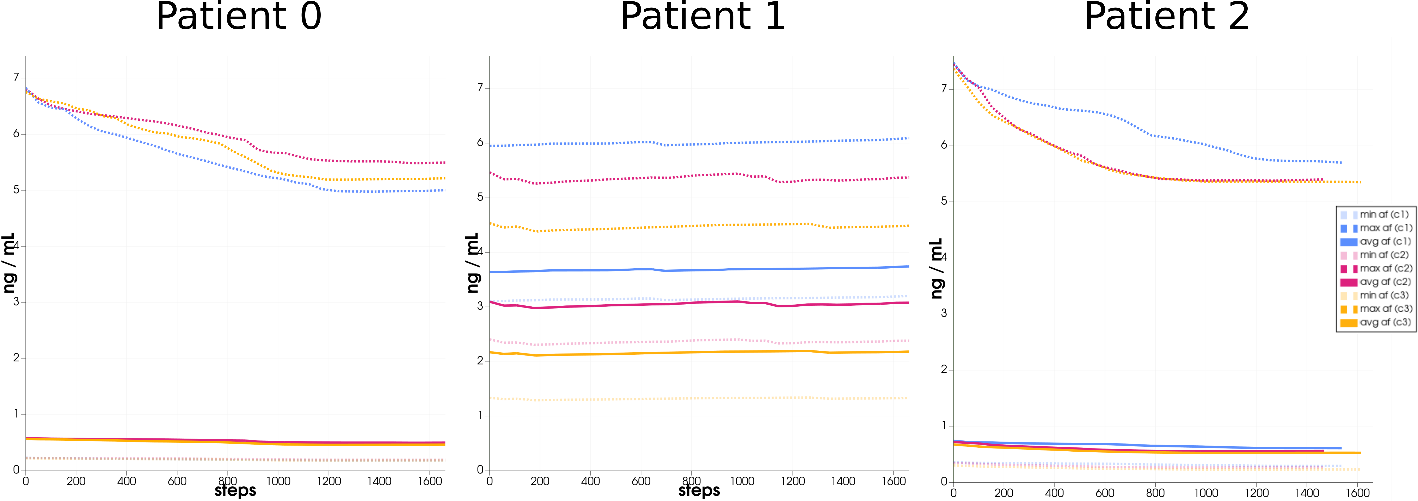


**Fig F**. **Average, maximum and minimum AF concentration for the first month of sprouting angiogenesis, for each patient and for each value of** $\boldsymbol{V}_{\boldsymbol{uc}}$**.** The $V_{pT}$ value is $47.3$ pg·mL^-1^·s^-1^**.** c1 (blue lines) correspond to $V_{uc}=2.3\cdot{10}^{-4}$ s^-1^, c2 (pink lines) to $V_{uc}=6.4\cdot{10}^{-4}$ s^-1^, and c3 (orange lines) to $V_{uc}=1.78\cdot{10}^{-3}$ s^-1^.


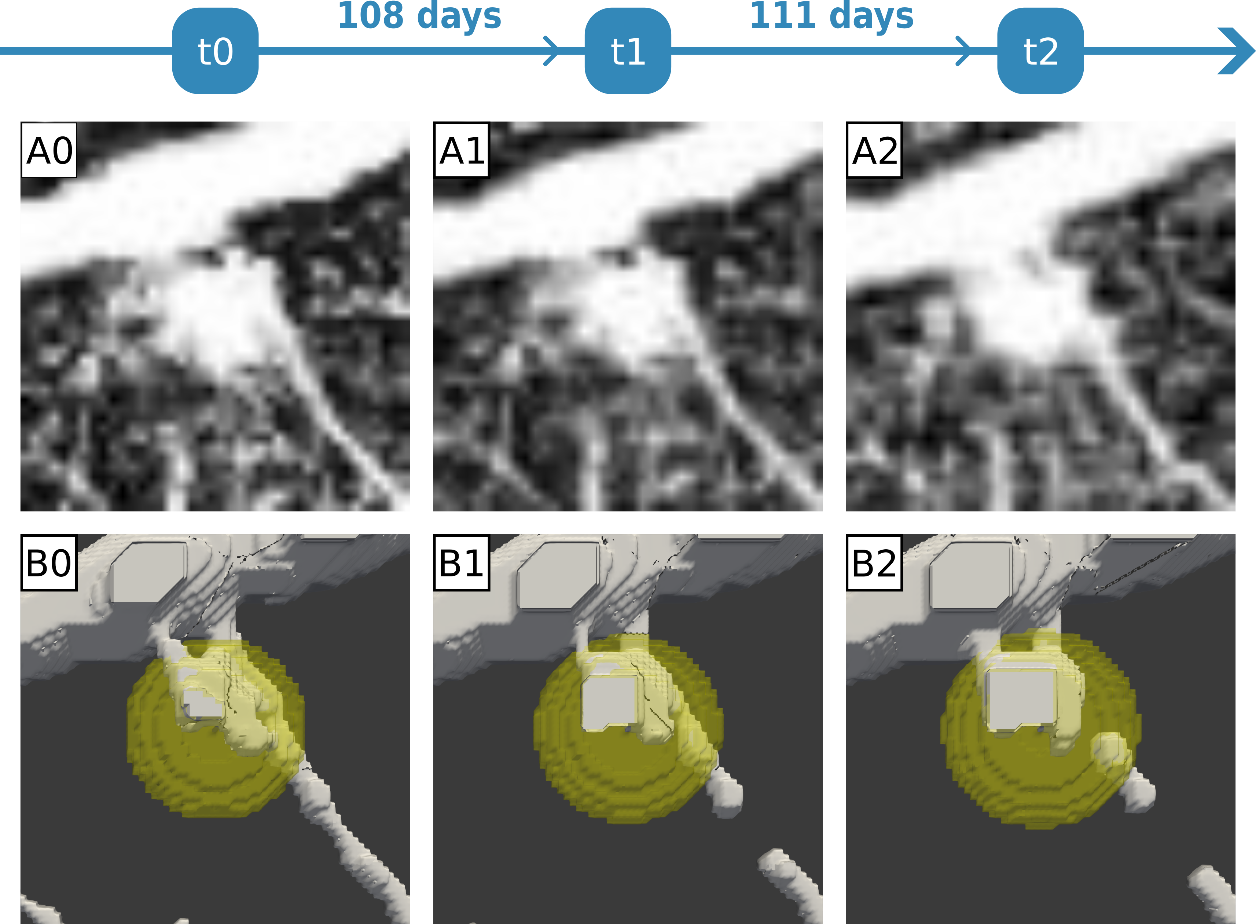


**Fig G**. **Simulation for 219 days of development using M = 10^-9^ mm^2^·s.** A0-2) OCTA images showing RH-induced capillaries in time for P1 (for a total of 219 days). B0-2) Result of the simulation for the same amount of time. Using $V_{pT}=47.3 pg\cdot\mathrm{mL}^{-1}\cdot s^{-1}$, $V_{uc}=2.3\cdot{10}^{-4} s^{-1}$, and $M={10}^{-9}$ mm^2^·s, the model recapitulates the stability of the vascular network inside the tumor, but not outside.


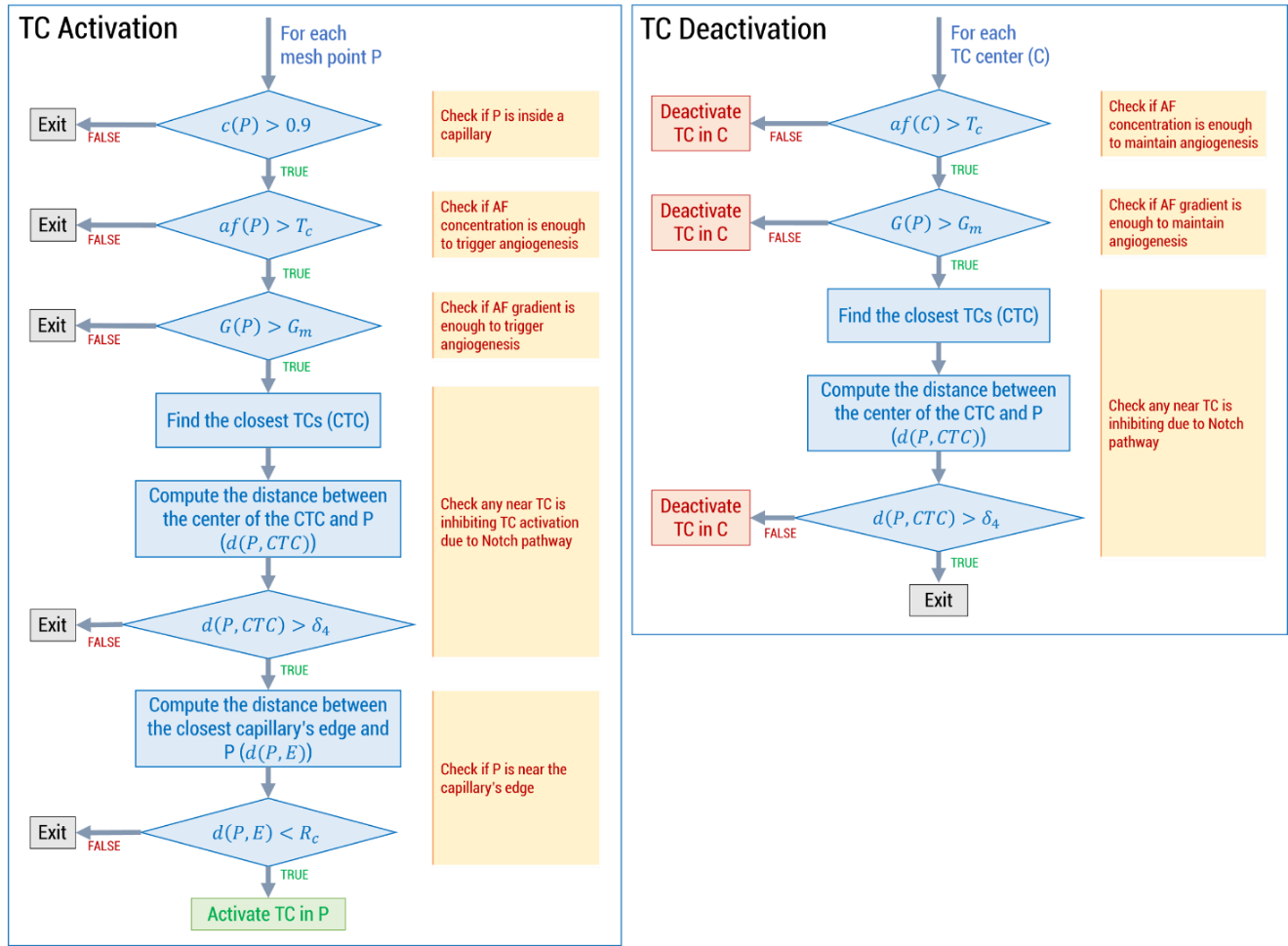


**Fig H. Algorithms regulating TCs’ activation (left) and deactivation (right).**


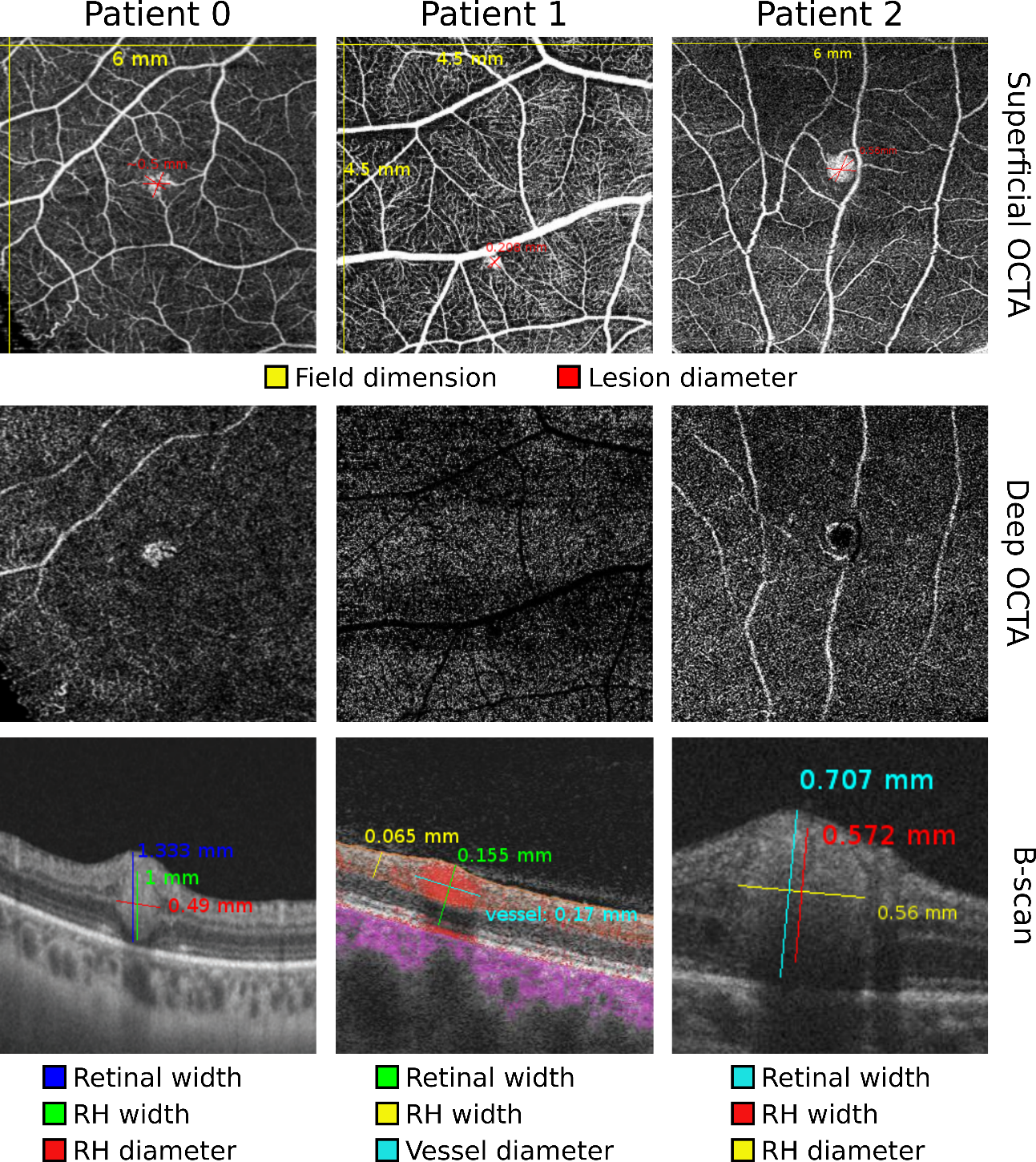


**Fig I. Annotated clinical images used to estimate the dimension and shape of the tumor.** We used the superficial OCTA (upper part of the image) to measure the diameter for each lesion and the B-scan (bottom part of the image) to measure the depth. We also measured the maximal retinal depth for each lesion to set the simulation box dimension. For patient 1, we assumed the lesion to be no deeper than the inner retinal layer, as the Deep OCTA (middle part of the lesion) showed no sign of abnormal vascularization. In the B-scan for patient 1, it is also evidenced the diameter of the vessel nearby the RH (the one above the lesion in the superficial OCTA).


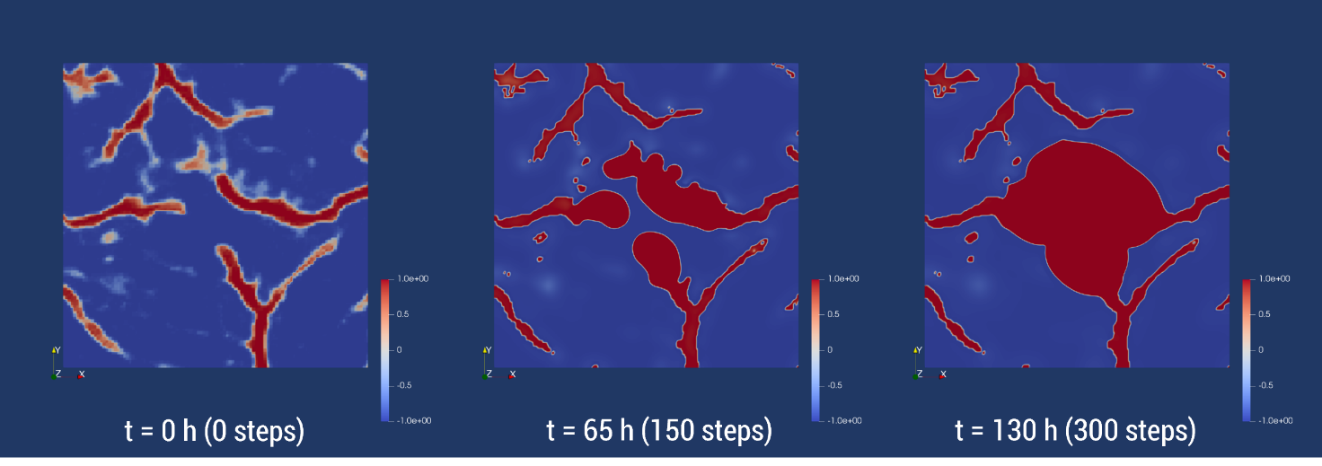


**Fig J. Simulation in 2D with** $\boldsymbol{\alpha}_{\boldsymbol{p}}\boldsymbol{=}\boldsymbol{\alpha}_{\boldsymbol{pSC}}$**, showing major capillaries enlargement after 130 hrs (300 steps)**. Since in the selected case report we cannot see any sign of enlargement, the model does not reproduce reality for this parameter choice.

**
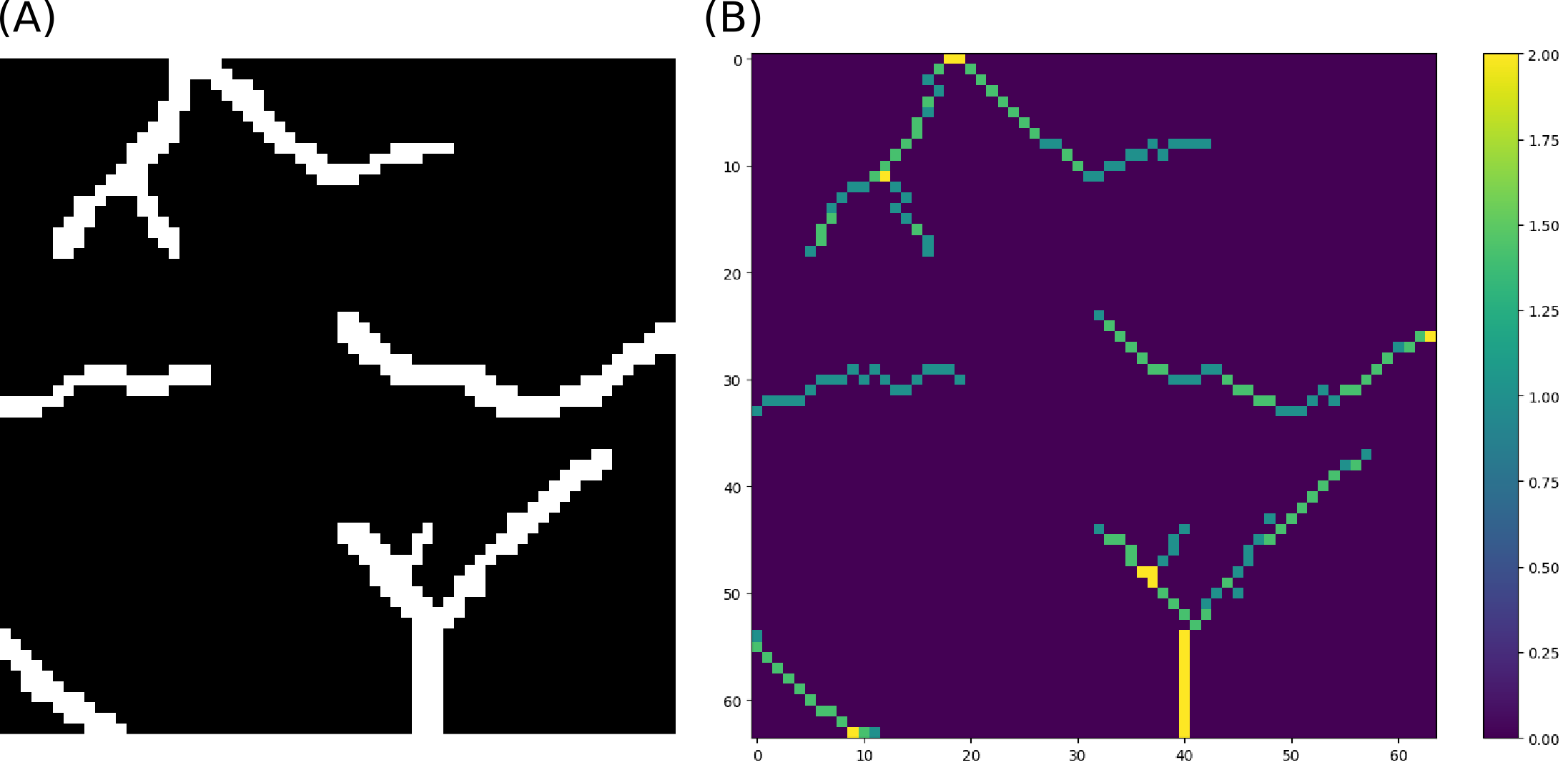
**

**Fig K. Comparison between segmentation (A) and distance transform (B) for the 2D-PICN of P0.** The distance is like a skeletonized image, where each line is reduced to 1 pixel width. However, each pixel above zero is equal to the local width of image (A). Notice that the higher values of the distance transform occur where the segmentation presents bolder vessels.


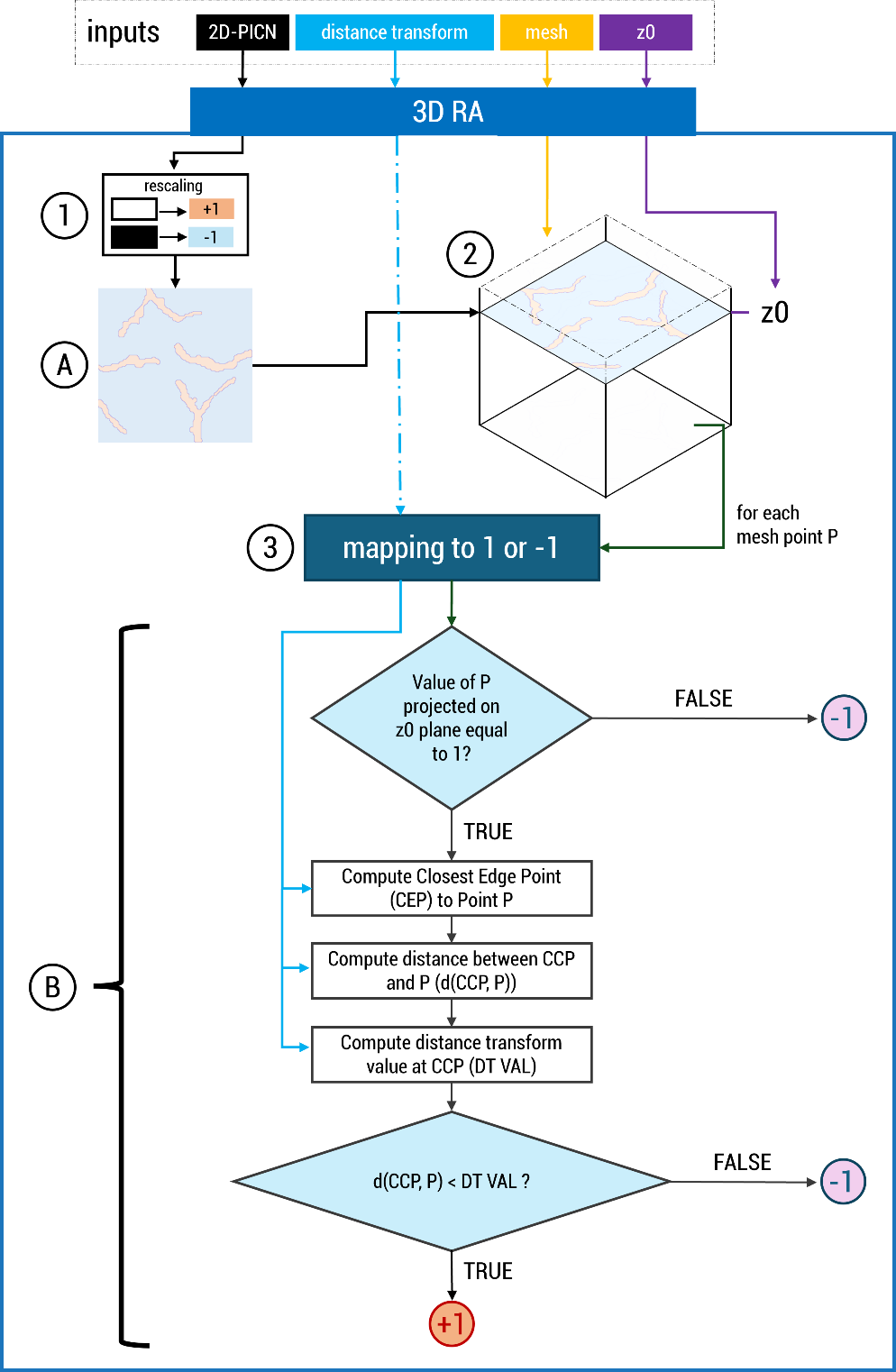


**Fig L. Schematic representation of our 3D RA.** On the top, we represented the inputs of the algorithms. (1) The first step rescales the 2D-PICN to obtain a 2D scalar field equal to 1 inside the capillaries and -1 otherwise (A). (2) The values of Matrix A are then mapped on the z0 plane of the 3D mesh. The user must specify the value of z0. (3) all other points are then mapped to 1 or -1 according to the procedure shown in the example. Notice that the algorithm employs the edges and the skeleton to compute the edge and center points.

| $\boldsymbol{D}_{\boldsymbol{af}}$ value | Reference |
| --- | --- |
| $\mathbf{10}^{\mathbf{-4}} \frac{\mathbf{m}\mathbf{m}^{\mathbf{2}}}{\mathbf{s}}$ | [20] |
| $\boldsymbol{1.16\cdot}\mathbf{10}^{\mathbf{-6}} \frac{\mathbf{m}\mathbf{m}^{\mathbf{2}}}{\mathbf{s}}$ | [21] |
| $\boldsymbol{2.22\cdot}\mathbf{10}^{\mathbf{-6}} \frac{\mathbf{m}\mathbf{m}^{\mathbf{2}}}{\mathbf{s}}$ | [22] |
| $\mathbf{10}^{\mathbf{-7}} \frac{\mathbf{m}\mathbf{m}^{\mathbf{2}}}{\mathbf{s}}$ | [23] |

**Table A. Some estimations of VEGF diffusivity reported in the literature.**

| **Patient** | **Lateral axis [μm]** | **Axial axis [μm]** | **Volume [mm^3^]** |
| --- | --- | --- | --- |
| P0 | 490 | 752 | 0.09 |
| P1 | 208 | 65 | 0.01 |
| P2 | 560 | 572 | 0.07 |

**Table B. Spatial dimension of each patient lesion.** The lateral axis is the diameter of the lesion parallel to the retinal layers, while the axial axis is perpendicular.

SI References

1. Kut C, Mac Gabhann F, Popel AS. Where is VEGF in the body? A meta-analysis of VEGF distribution in cancer. Br J Cancer 2007 977. 2007;97: 978–985. doi:10.1038/sj.bjc.6603923

2. Los M, Aarsman CJM, Terpstra L, Wittebol-Post D, Lips CJM, Blijham GH, et al. Elevated ocular levels of vascular endothelial growth factor in patients with von Hippel-Lindau disease. Ann Oncol. 1997;8: 1015–1022. doi:10.1023/A:1008213320642

3. Travasso RDM, Poiré EC, Castro M, Rodrguez-Manzaneque JC, Hernández-Machado A. Tumor angiogenesis and vascular patterning: A mathematical model. PLoS One. 2011;6: e19989. doi:10.1371/journal.pone.0019989

4. Na X, Wu G, Ryan CK, Schoen SR, Di’Santagnese PA, Messing EM. Overproduction of vascular endothelial growth factor related to von Hippel-Lindau tumor suppressor gene mutations and hypoxia-inducible factor-1α expression in renal cell carcinomas. J Urol. 2003;170: 588–592. doi:10.1097/01.ju.0000074870.54671.98

5. Ye DY, Bakhtian KD, Asthagiri AR, Lonser RR. Effect of pregnancy on hemangioblastoma development and progression in von Hippel-Lindau disease: Clinical article. J Neurosurg. 2012;117: 818–824. doi:10.3171/2012.7.JNS12367

6. Vasudev NS, Reynolds AR. Anti-angiogenic therapy for cancer: Current progress, unresolved questions and future directions. Angiogenesis. 2014;17: 471–494. doi:10.1007/S10456-014-9420-Y

7. Stokes CL, Lauffenburger DA, Williams SK. Migration of individual microvessel endothelial cells: stochastic model and parameter measurement. 1991.

8. Blinder YJ, Freiman A, Raindel N, Mooney DJ, Levenberg S. Vasculogenic dynamics in 3D engineered tissue constructs. Sci Rep. 2015;5: 1–8. doi:10.1038/srep17840

9. Guedez L, Rivera AM, Salloum R, Miller ML, Diegmueller JJ, Bungay PM, et al. Quantitative Assessment of Angiogenic Responses by the Directed in Vivo Angiogenesis Assay. Am J Pathol. 2003;162: 1431. doi:10.1016/S0002-9440(10)64276-9

10. Harper K, Yatsyna A, Charbonneau M, Brochu-Gaudreau K, Perreault A, Jeldres C, et al. The Chicken Chorioallantoic Membrane Tumor Assay as a Relevant In Vivo Model to Study the Impact of Hypoxia on Tumor Progression and Metastasis. MDPI Cancers. 2021;13: 1093. doi:10.3390/CANCERS13051093

11. Shamloo A, Ma N, Poo MM, Sohn LL, Heilshorn SC. Endothelial cell polarization and chemotaxis in a microfluidic device. Lab Chip. 2008;8: 1292–1299. doi:10.1039/B719788H

12. Barkefors I, Le Jan S, Jakobsson L, Hejll E, Carlson G, Johansson H, et al. Endothelial Cell Migration in Stable Gradients of Vascular Endothelial Growth Factor A and Fibroblast Growth Factor 2: EFFECTS ON CHEMOTAXIS AND CHEMOKINESIS. J Biol Chem. 2008;283: 13905–13912. doi:10.1074/JBC.M704917200

13. Milo R, Jorgensen P, Moran U, Weber G, Springer M. BioNumbers—the database of key numbers in molecular and cell biology. Nucleic Acids Res. 2010;38: D750. doi:10.1093/NAR/GKP889

14. Song M, Finley SD. Mechanistic characterization of endothelial sprouting mediated by pro-angiogenic signaling. Microcirculation. 2022;29: e12744. doi:10.1111/MICC.12744

15. Kihara T, Ito J, Miyake J. Measurement of Biomolecular Diffusion in Extracellular Matrix Condensed by Fibroblasts Using Fluorescence Correlation Spectroscopy. PLoS One. 2013;8: 82382. doi:10.1371/JOURNAL.PONE.0082382

16. Bateman A, Martin MJ, Orchard S, Magrane M, Agivetova R, Ahmad S, et al. UniProt: the universal protein knowledgebase in 2021. Nucleic Acids Res. 2021;49: D480–D489. doi:10.1093/NAR/GKAA1100

17. Finley SD, Dhar M, Popel AS. Compartment model predicts VEGF secretion and investigates the effects of VEGF Trap in tumor-bearing mice. Front Oncol. 2013;3 JUL: 196. doi:10.3389/fonc.2013.00196

18. Vempati P, Popel AS, Gabhann F Mac. Extracellular regulation of VEGF: isoforms, proteolysis, and vascular patterning. Cytokine Growth Factor Rev. 2014;25: 1–19. doi:10.1016/j.cytogfr.2013.11.002

19. Sagar P, Rajesh R, Shanmugam M, Konana VK, Mishra D. Comparison of optical coherence tomography angiography and fundus fluorescein angiography features of retinal capillary hemangioblastoma. Indian J Ophthalmol. 2018;66: 872–876. doi:10.4103/IJO.IJO_1199_17

20. Lai X, Friedman A. Mathematical modeling in scheduling cancer treatment with combination of VEGF inhibitor and chemotherapy drugs. J Theor Biol. 2019;462: 490–498. doi:10.1016/J.JTBI.2018.11.018

21. Guerra A, Belinha J, Mangir N, MacNeil S, Natal Jorge R. Sprouting Angiogenesis: A Numerical Approach with Experimental Validation. Ann Biomed Eng. 2021;49: 871–884. doi:10.1007/S10439-020-02622-W/TABLES/1

22. Phillips CM, Lima EABF, Woodall RT, Brock A, Yankeelov TE. A hybrid model of tumor growth and angiogenesis: In silico experiments. PLoS One. 2020;15: e0231137. doi:10.1371/JOURNAL.PONE.0231137

23. Levine HA, Pamuk S, Bölümü M, Sleeman BD, Nilsen-Hamilton M, Cotran RAS, et al. Mathematical modeling of capillary formation and development in tumor angiogenesis: Penetration into the stroma. Bull Math Biol 2001 635. 2001;63: 801–863. doi:10.1006/BULM.2001.0240
