# Supplementary material for: Mathematical modeling and simulation of tumor-induced angiogenesis in retinal hemangioblastoma
Source: PLoS Comput Biol. 2025 Sep 11;21(9):e1012799. doi: 10.1371/journal.pcbi.1012799 (PMC12503348; doi:10.1371/journal.pcbi.1012799)
Supplement: Attachment — Submitted filename: Response to Reviewers.docx [file pcbi.1012799.s002.docx]

Dear reviewers and editors,

We thank you for carefully reading our manuscript and for sharing your constructive comments on our work. We modified the manuscript following the reviewer’s suggestions. Below, you can find our answers to each of the comments.

We hope that our manuscript now matches the standards of PLOS Computational Biology.

All the best,

The authors

# Answer to Reviewer #1

**Comment 1: Some claims are overstated. For example, “proving” should be “supporting” (p. 3, line 8), “an exceptional” should be “a useful” (p. 5, line 8), “recapitulate” (abstract) and “reproduces” (p. 12, line 1) are too strong.**

In agreement with this recommendation, we modified some of the terms we used in the manuscript. We changed “proving” (p.3, line 8) with “supporting”, and “an exceptional” (p.5, line 8) with “a useful”. We further substituted “recapitulate” in the abstract with “simulate.” Finally, we changed the title of the second section of the results from “The Phase-Field Model reproduces the vascular sprouting for each patient” to “The simulations in simplified settings present vascular sprouting and dense capillary networks.” We believe this new title clarifies what we meant by “reproduces” in the previous title.

**Comment 2: P. 11. Diffusivity should have units of mm^2 s^(-1).**

We apologize for the typo. We corrected the units of measure in the diffusivity.

**Comment 3: P. 17. A volume of 0.001 mm3 corresponds to a radius of 62 micron. A volume of 0.01 mm3 corresponds to a radius of 134 micron. The values given are not consistent.**

Here the problem is that we were not enough clear regarding the method used to compute the volumes. They were not derived from the diameters using geometric formulas (e.g., for spheres or ellipsoids), but rather obtained from the volume percentages reported in the upper section of Figure 3 (now Figure 2, see below). The confusion may stem from our use of the term 'converting,' which we used to imply that the reported volumes correspond to the values observed for each patient. To clarify this point, we have changed the following sentence:

“Converting these diameters into RH volumes, we get 0.01 mm^3^ for P0, 0.001 mm^3^ for P1, and 0.006 mm^3^ for P2”

With:

“We observe a similar pattern for the tumor volumes. Specifically, the onset of sprouting angiogenesis occurs at a minimal volume corresponding to 6% of the maximum for patients P0 and P2, and 72% for P1. In absolute terms, these thresholds translate to 0.01 mm³ for P0, 0.001 mm³ for P1, and 0.006 mm³ for P2.”

We hope the revised wording clarifies how we derived the minimum volume for each patient, and we apologize for any confusion caused.

**Comment 4: P. 27. The methods of the model are not clearly and consistently presented. On p. 29, should be “Cahn-Hilliard.” The justification for this equation is not well explained. It is stated that “The agent-based algorithm handles … stalk cells’ proliferation.” According to Travasso et al. (2011), however, stalk cell proliferation is governed by the Cahn-Hilliard equation. The overall rationale for the phase-field model should be better explained in this section. An agent-based model is used to predict the occurrence of tip cells. Apparently, tip cells become active when a sufficiently high growth factor level and gradient are present near existing stalk cells, and the point is not too close to an existing tip cell. The region of stalk cells (c > 0) expands in response both to the effects of growth factors and to the updating of the value of c in the vicinity of tip cells. The Cahn-Hilliard model provides a method to generate a smooth function whose level set c = 0 defines the boundary of capillaries. An explanation along these lines in the main text would be helpful. It is not clear why equation 3 for growth factor diffusion is given in the Results (p. 7) and not in this section.**

We apologize for the lack of clarity and for the typo with “Cahn-Hilliard.” We did not provide a detailed justification for each term in the model because the equations in the Methods section are extensively discussed in Travasso et al. (2011). However, we acknowledge that further explanation could improve the clarity of the manuscript. Splitting the model description between the Methods and Results sections may have introduced additional confusion. Our intent was to emphasize the differences between our modified equations (e.g., for tumor evolution and angiogenic factor distribution) and those in the original Travasso model, but we now recognize this structure may not be optimal.

Regarding stalk cell (SC) proliferation, while Travasso et al. model SC dynamics and proliferation using the Phase-Field equation, our interpretation is different. SCs, by definition, are located near tip cells (TCs) during capillary sprouting, and endothelial cells exhibit increasingly mature phenotypes the farther they are located from the nearest TC [1]. The original proliferation term (Bp(af)·c·H(c)) applies uniformly across the vascular network and does not capture this spatial dependence. Therefore, we introduced a modification: we used the original term to model the proliferation of mature endothelial cells throughout the domain and added the term Sp to represent SC proliferation localized near TCs. As shown in Fig. S10, this adjustment yields more realistic vascular sprouting than the original model. We acknowledge, however, that we did not explain these modifications with sufficient clarity in the manuscript.

To address this comment and make the model presentation clearer, we introduced the following changes to the manuscript:

**Change 1**: We corrected “Cahn-Hilliard”.

**Change 2**: We moved the entire model description in the Methods section, to a subsection called “Mathematical Model”. For further clarity, this subsection is divided into three parts: “Tumor growth” (equations for RH progression); “Tumor-induced angiogenesis” (vascular sprouting model, based largely on Travasso et al., 2011); “Angiogenic factors distribution”  (reaction-diffusion equation for AF dynamics). We also moved Figure 1 (now Figure 5) and Table 1 together with this section.

**Change 3**: To clarify the role of each term in the Phase-Field equation employed for c, we changed the following sentence (previously on p.29, line 6):

“Where the first term is a Cahn-Hilliard term accounting for the interface dynamics, and the second term accounts for endothelial cells’ proliferation.”

With:

“The first term of the equation represents a Cahn–Hilliard component, which models the morphology of the capillaries and defines the interface between the blood vessels and the surrounding tissue. This term constrains the field variable c to attain values close to 1 within the blood vessels and approximately –1 in the adjacent tissue, maintaining a diffuse interface between the two regions of the tumor microenvironment. Furthermore, it ensures the evolution of a smooth and physiologically realistic vessel–tissue boundary over time. The second term accounts for the proliferation of endothelial cells driven by AFs.”

**Change 4**: To clarify the algorithm regulating the activation, motion, and deactivation of TCs, we modified the following sentence (previously on p.29, line 10):

“The agent-based algorithm handles TCs’ activation, deactivation, motion, and stalk cells’ (SC) proliferation.”

With:

“The agent-based algorithm handles TCs’ activation, deactivation, motion, and stalk cells (SC) proliferation. This method is described in detail in the original publication by Travasso et al. (20). For completeness, we provide a concise summary of its key principles. The algorithm places a new tip cell (TC) at each mesh point within the vascular network—defined by locations where the field variable c is greater than or equal to 1—and where both the concentration and gradient of the angiogenic factor (AF) exceed specified thresholds, denoted Tc and Gm, respectively. To account for the inhibitory effect of the Delta-Notch signaling pathway between neighboring endothelial cells, the algorithm ensures that no two TCs are positioned closer than a minimum distance δ4. Additionally, any TC is deactivated if its local conditions no longer satisfy the above criteria. Finally, the algorithm governs TC migration, with cells advancing along the AF gradient according to the following velocity law:

Equation 5

$$v=\left\{ \begin{matrix} \chi\frac{\nabla af}{G} & \mathrm{if}G_{m}\leq G<G_{M} \\ \chi\frac{\nabla af}{G}G_{M} & if G\geq G_{M} \end{matrix} \right.$$

Where G is the norm of *∇af*. A detailed schematic of the algorithm is depicted in Fig. S8.”

Equation 5 before was presented in the Supplementary Information, together with Fig. S8. We hope the current presentations will make the model more understandable.

**Change 5**: To clarify the difference between SCs proliferation in our model with the original Travasso model, we changed the following sentence (previously on p. 29, line 19):

“The original model did not include this distinction, and we added it to differentiate the SCs’ proliferation from mature endothelial cells’ proliferation (see SI for details).”

With:

“We highlight that the original model did not include this distinction, and it assumed that stalk cells proliferation occurs through the proliferation term In Equation 6. However, this choice does not consider that TCs are present only in the immediate surroundings of the TCs, and that mature endothelial cells are much less proliferative than SCs (52). Indeed, simulating the model without this distinction led us to unrealistic enlargement of all the capillaries in the proximity of the tumor, regardless of the presence or the proximity of TCs (see Section 2 of SI and Fig. S10). “

Notice that reference (52) in the manuscript is [1] (Cuervo H et al. 2023).

**Comment 5: P. 30. The bisection method is indeed well-known and does not need to be explained. Delete text “Given two … the minimal volume.” Also delete text “If dt_max is bigger … can occur” on pp. 31-32.**

Following the reviewer’s suggestion, we changed the following paragraph:

“To efficiently find such minimal size, we took inspiration from the well-known bisection method. Given two tumor volumes, one inducing TC activation (V_pos_, initially 100%) and one not (V_neg_, initially 0%), we pick the mean between the two values, and we assess TC activation for that value (V_half_, initially, 50%). If we observe TC activation at V_half_, the minimal volume must be between V_neg_ and V_half_. Else, the minimal volume must be between V_pos_ and V_half_. So, we set the new V_half_ value equal to the mean of the two extremes of the interest interval, and we iteratively compute more precises estimates of the minimal volume. The algorithm stops when the difference between V_pos_ and V_neg_ is less than 5% of the maximum tumor volume.”

In a more concise form:

“To efficiently find such minimal size, we used the bisection method. The iteration stops when the change between successive volume estimates is less than 5% of the maximum tumor volume.”

We introduced a similar change to the part of the text regarding dt_max_ and dt_min_. We modified:

“To prevent such bias, every time a dt higher than 1 is employed, we also checked if any TC could activate earlier in the given time span. We employed an algorithm inspired from the bisection method, similarly to the one explained earlier for the estimation of the minimal tumor volume. If dtmax is bigger than 1, we solve the PDEs system and we check if the conditions for TC activation are met anywhere in the domain. If the test is negative, we update the simulation state to dtmax and we assume that no TC activated in that time span. Else, we solve again the system for dthalf = dtmax / 2 and we check if TC activation occurs. If TC activation occurs at dthalf, it means that the vascular sprouting could occur in the time span [0, dthalf]. Else, it means that the sprouting could have occurred earlier, in [dthalf, dtmax]. So, we compute the new dthalf as the mean of the extremes of the new time span, and we iteratively find the minimum dt value for which TC activation can occur. The algorithm stops when the difference between the extremes of the interest interval is less than dtmin.”

In the following way:

“To prevent such bias, every time a *dt* higher than 1 is employed, we also checked if any TC could activate earlier in the given time span. To do so, we again employed the bisection method to find a dt value at which a TC activation could occur. The algorithm stops when the difference between two estimates of such dt value is smaller than dt_min_.”

**Comment 6: SI, p. 4, should be M=10^(-15) m2 s^(-1).**

We apologize for the typo. We corrected the number in the SI.

# Answer to Reviewer #2

**Comment 1: In page 5 the authors indicate that CMMs are an exceptional tool for cancer research. Then they indicate that CMMs allow for the interplay between complex biological phenomena and for the integration of patient-specific data. However, the only reference the authors introduce here is 15 years old and does not discuss the use of these models in precision medicine. So, recent references are missing. Also, in the next paragraph, the authors start by saying that there are different kinds of CMMs that exploit different data and (use) different mathematical tools. However they just discuss PFMs. Therefore, I also suggest that the authors should include a paragraph on other common methods besides PFMs.**

We agree with the reviewer that we provided insufficient citations to highlight the relevance of CMMs in cancer biology and clinical research. To make our statement more consistent, we added the following citations:

16. Altrock PM, Liu LL, Michor F. The mathematics of cancer: integrating quantitative models. Nat Rev Cancer 2015 1512 . 2015 Nov 24.
17. Strobl MAR, Gallaher J, Robertson-Tessi M, West J, Anderson ARA. Treatment of evolving cancers will require dynamic decision support. Ann Oncol. 2023 Oct 1;34(10):867–84.
18. Lorenzo G, Ahmed SR, Hormuth II DA, Vaughn B, Kalpathy-Cramer J, Solorio L, et al. Patient-Specific, Mechanistic Models of Tumor Growth Incorporating Artificial Intelligence and Big Data. Annu Rev Biomed Eng. 2024 Apr 9.
19. McDonald TO, Cheng Y-C, Graser C, Nicol PB, Temko D, Michor F. Computational approaches to modelling and optimizing cancer treatment. Nat Rev Bioeng 2023 110. 2023 Jul 19.
20. Wölfl B, te Rietmole H, Salvioli M, Kaznatcheev A, Thuijsman F, Brown JS, et al. The Contribution of Evolutionary Game Theory to Understanding and Treating Cancer. Dyn Games Appl 2021 122. 2021 Aug 30.

All the citations we added are recent and discuss the application of mathematical models in cancer, including precision medicine.

Regarding the different techniques used in mathematical oncology, a comprehensive discussion on each technique would be out of scope, especially when excellent reviews on the subject (e.g., the references we added) are available. However, we decided to mention the major techniques employed. More precisely, we changed the following sentence:

“There are different kinds of CMMs, exploiting different data and mathematical tools. Phase Field Models (PFMs) are based on partial differential equations (PDEs) describing the evolution in time of the boundaries of biological tissues, including neoplasms (16)”

With:

“Among the most commonly employed techniques are Ordinary Differential Equations (ODEs), Partial Differential Equations (PDEs), stochastic processes, agent-based models, control theory, and game theory—each contributing uniquely to the modeling and understanding of cancer dynamics (see (19) and (20) for a comprehensive overview). Within this framework, Phase-Field Models (PFMs) represent a specific class of PDE-based methods that capture the spatiotemporal evolution of biological tissue interfaces, including neoplasms (21)”

Notice that we mentioned some of the references we added in this section.

**Comment 2: Many vessels are missing in the segmentation. The vasculature should have a vessel density of around one capillary per 20 µm, but there are only represented the thicker vessels around a very large half a millimetre size tumour. It can be clearly seen that vessels are missing because the vessels represented have dead-ends. The vessel structure is particularly important since the main results hinge on the balance between the tumour-production and capillary-uptake of AFs. The mechanism suggested by the simulation is that angiogenesis will happen until sufficient vessels are present to capture the VEGF produced by the tumour, or to replace a sufficiently large number of VEGF-producing tumour cells. If this is the case, then the vessels and the AF vessel uptake should be exceedingly well simulated, and that is not the case. The authors should discuss the shortcomings carefully. What would the model predict to the neo-vascularisation in the situation where all the vessels were taken into consideration? I.e, can the authors extend the simulation by building an artificial dense vessel network that connects to the thick represented vessels and that perfuses the tissue, and then run the simulation on that dense network, to see if the main conclusions still hold (i.e. that angiogenesis will stop whenever there are sufficient vessels to uptake the tumor-produced VEGF)?**

Thank you for your thoughtful feedback. One of the key limitations of our study is indeed the choice of the initial capillary network. Due to the lack of images before tumor onset, we segmented the major vessels in the tumor vicinity that could be clearly distinguished from the dense plexus induced by RH development. This choice is already inherently biased and is meant to be a realistic representation of the initial condition in the absence of a real clinical image to use. On the top of that, the manual segmentation also induces biases. For instance, it’s true that we did not segment some of the smaller vessels in the surrounding of the tumour, because many of them are not clearly visible in their full length. To mitigate these risks, our segmentation was conducted in collaboration with expert ophthalmologists with extensive clinical experience in the disease (our coauthors, Dr. Venkatesh and Dr. Azad) [5–7].

This constitutes a significant limitation of our work, but we believe it does not impact the validity of our considerations. Angiogenesis, particularly in tumor contexts, is known to be driven by the balance of angiogenic factors such as VEGF. This mechanism is well-established and not a novel outcome of our model [2]. Our model simulates this balance by a dynamic interaction between AF production by RH (in agreement with the observations reported in the introduction of the manuscript, e.g. [3-4]) and AF transport and decay. As with every mathematical or computational model, it represents a simplification of a complex biological phenomenon, but it's coherent with the pathology of the tumor.

The rapid stabilization of capillary growth observed in our simulations primarily results from two elements: the velocity of the endothelial tip cells—which is a model parameter for which the value strongly agrees with experimental data—and the reduction in angiogenic factor (AF) concentration due to uptake by the newly formed vasculature. As shown in Supplementary Figure S6, this decrease in AF concentration is relatively modest. This suggests that once the AF concentration and its gradient exceed the threshold required to initiate vascular sprouting (which has also been derived from experimental evidence), the process proceeds and rapidly self-limits as the network reaches a stable configuration.

It is plausible that a denser pre-existing vascular network would necessitate a larger tumor mass to achieve a comparable level of AF sufficient for sprouting, implying that our estimated minimal tumor volumes might be underestimated. Nonetheless, it is noteworthy that the tumor in Patient 1 (P1) has a diameter of 208 μm and already exhibits vascularization. This observation supports the notion that if a critical minimum diameter for vascularization exists, it likely falls within the range we have identified.

In conclusion, we recognize that these considerations were not fully articulated in the manuscript. Accordingly, we have revised the "Initial capillary network" section in the Methods to clarify our rationale:

“Since there was no record of the shape of the capillary network before tumor development for any patient, we had to construct an image representing a putative initial capillary network (PICN) to run our simulations. We derived our initial conditions from manual segmentations conducted in collaboration with two expert ophthalmologists, selecting the major vessels in the surroundings of the tumor and excluding those induced by the tumor. At this early stage, it is easy to distinguish between the tumor-induced vessels and the vessels already present before tumor development. Moreover, choosing to derive our PICN from the surroundings of the tumor allowed us to compare more easily or simulations with the clinical images. The manual segmentation were conducted using the Fiji (47) plugin LabKit (48).”

Also, we included a section in the discussion to highlight this limitation. The last part of the discussion was modified as follows:

“In summary, our model demonstrates a remarkable capacity to reproduce the key features of retinal hemangioblastoma (RH) pathology and its temporal development—features that are otherwise difficult to capture in the absence of an appropriate animal model. These findings indicate that the model holds potential for predicting RH progression and evaluating therapeutic interventions in a patient-specific manner.

Nonetheless, the study presents several limitations that warrant consideration. The most significant constraint lies in the selection of the initial conditions for the simulations. Specifically, we were unable to acquire OCTA images of the vascular network prior to tumor onset and angiogenesis. Consequently, the initial vascular structures were derived from manually segmented images of prominent vessels that could be clearly distinguished around the tumor and appeared unrelated to RH-induced angiogenesis. This reliance on a realistic—but not patient-specific—initial condition introduces a potential source of bias. Moreover, the manual segmentation process itself may introduce subjective variability. To mitigate these limitations, initial conditions were chosen in collaboration with two expert ophthalmologists with established expertise in RH. Still, alternative plausible initial conditions could be considered.

Importantly, the consistency of our results across three different patients—with distinct vascular architectures and tumor morphologies—strongly supports the robustness of our main findings against variations in the initial conditions. However, further validation involving a larger dataset of OCTA images and longitudinal observations across multiple time points is essential to rigorously assess the predictive capability of the phase-field model.

Another limitation pertains to the computational demands of simulating tumor progression beyond one month. This constraint currently restricts the temporal scope of our simulations. Nevertheless, the integration of advanced numerical solvers and adaptive mesh refinement techniques (42) may substantially enhance computational efficiency, thereby enabling long-term simulations over multiple years.

Overall, our study provides novel insights into RH pathophysiology and treatment response, while also underscoring the value of OCTA imaging as a tool for studying angiogenesis in human subjects through the lens of mathematical modeling.”

We hope that these adjustments help to clarify the rationale in the selection of the initial conditions and its inherent limits.

**Minor point 1) Page 6, line 8. "...are both hard to observe...". Should replace "observe" for "quantify". With the word "observe", the sentence can be read as to say that hypoxic produced AFs are uncommon to exist in the clinical setting.**

Thanks for the recommendation. We changed the text accordingly.

**Minor point 2) Typesetting: in the text, the variables should be in italic.**

We set all the variables in the text to italic. We also noticed that there were some inconsistencies in the text: sometimes the variables appeared formatted as equation and sometimes as normal text. We homogenized everything to normal text in italic, leaving the equation typesetting only for the equations presented in Methods.

**Minor point 3) Page 8: replace several instances of "from" with "by". ("uptake by the capillaries" and not "uptake from the capillaries")**

Thanks for the note. We fixed the text accordingly.

**Minor point 4) Figs 2 and 4, scale missing in the simulation. Figs 4 E0, E1 and E2: very small letters and numbers; very hard to read.**

We added the spatial scale to Fig. 2 (now Fig. 1). We also agree on the fact that the legends in Fig. 4 (now Fig. 3) appeared very small. We set a unique legend on the top of Fig3E0-2.

# References

1. Cuervo, H., Mühleder, S., García-Gónzalez, I. & Benedito, R. Notch-mediated cellular interactions between vascular cells. Curr. Opin. Cell Biol. 85, 102254 (2023).
2. Dudley, A. C., & Griffioen, A. W. (2023). Pathological angiogenesis: mechanisms and therapeutic strategies. Angiogenesis, 26(3), 313–347. <https://doi.org/10.1007/s10456-023-09876-7>
3. Chan C-C, Collins ABD, Chew EY. Molecular Pathology of Eyes with von Hippel–Lindau (VHL) Disease: A Review. Retina [Internet]. 2007 Jan [cited 2021 Jul 26];27(1):1. Available from: /pmc/articles/PMC1971131/
4. Los M, Aarsman CJM, Terpstra L, Wittebol-Post D, Lips CJM, Blijham GH, et al. Elevated ocular levels of vascular endothelial growth factor in patients with von Hippel-Lindau disease. Ann Oncol. 1997 Oct 1;8(10):1015–22.
5. Goswami A, Surve A, Venkatesh P. Optical Coherence Tomography Angiography of Early Stage 1a Retinal Hemangioblastoma in Von-Hippel-Lindau. J Kidney Cancer VHL. 2021 Sep 23;8(3):15-18. doi: 10.15586/jkcvhl.v8i3.158. PMID: 34631390; PMCID: PMC8476344.
6. Azad SV, Takkar B, Venkatesh P, et al. Swept source: optical coherence tomography angiography features of choroidal osteoma with choroidal neovascular membrane. Case Reports 2016;2016:bcr2016215899.
7. Venkatesh, P. (2023). *Handbook of Vitreoretinal surgery*. CRC Press.
